# Transcriptomic analysis reveals new hippocampal gene networks induced by prolactin

**DOI:** 10.1038/s41598-019-50228-7

**Published:** 2019-09-24

**Authors:** Erika Alejandra Cabrera-Reyes, América Vanoye–Carlo, Mauricio Rodríguez-Dorantes, Edgar Ricardo Vázquez-Martínez, Nadia Alejandra Rivero-Segura, Omar Collazo-Navarrete, Marco Cerbón

**Affiliations:** 1 Unidad de Investigación en Reproducción Humana, Instituto Nacional de Perinatología-Facultad de Química, Universidad Nacional Autónoma de México. CDMX, México, 04510 Mexico; 2 Laboratorio de Neurociencias, Instituto Nacional de Pediatría, SS. CDMX, México, 04530 Mexico; 3 Instituto Nacional de Medicina Genómica, CDMX, México, 14610 Mexico; 4 Laboratorio Nacional de Recursos Genómicos, Instituto de Investigaciones Biomédicas, Universidad Nacional Autónoma de México, CDMX, México, 04510 Mexico; 5 División de Ciencias Básicas, Instituto Nacional de Geriatría, CDMX, México, 10200 Mexico

**Keywords:** Transcriptomics, Molecular neuroscience

## Abstract

Prolactin (Prl) is a pleiotropic hormone with multiple functions in several tissues and organs, including the brain. In the hippocampus, Prl has been implicated in several functions, including neuroprotection against excitotoxicity in lactating rats and in Prl-treated ovariectomized animals. However, the molecular mechanisms involved in Prl actions in the hippocampus have not been completely elucidated. The aim of this study was to analyse the hippocampal transcriptome of female Prl-treated ovariectomized rats. Transcriptomic analysis by RNASeq revealed 162 differentially expressed genes throughout 24 h of Prl treatment. Gene Ontology analysis of those genes showed that 37.65% were involved in brain processes that are regulated by the hippocampus, such as learning, memory and behaviour, as well as new processes that we did not foresee, such as glial differentiation, axogenesis, synaptic transmission, postsynaptic potential, and neuronal and glial migration. Immunodetection analysis demonstrated that Prl significantly modified microglial morphology, reduced the expression of Cd11b/c protein, and altered the content and location of the neuronal proteins Tau, Map2 and Syp, which are involved in axogenic and synaptic functions. This novel delineation of Prl activity in the hippocampus highlights its importance as a neuroactive hormone, opens a new avenue for understanding its actions and supports its participation in neuronal plasticity of this brain area.

## Introduction

Prolactin is a pleiotropic hormone in vertebrates with more than 300 described functions that fall into five major categories: reproduction, brain processes and behaviour, growth and development, water and electrolyte balance, and immunomodulation^1^. The expression of *Prl* and its receptor have been detected at the transcriptional level in several brain areas, such as the olfactory bulb, corpus callosum, choroid plexus, amygdala, hypothalamus, thalamus, cerebral cortex and hippocampus^2,3^.

Physiological behaviours regulated by the hippocampus are complex and implicated in several brain processes, including storage and consolidation of spatial and declarative memory and acquisition and retrieval of information^4^. In this regard, Prl improves memory, cognition and learning^5–8^. In addition, Prl induces several processes, such as neurogenesis, neuronal proliferation and survival^9–11^, neuroplasticity and dendritic remodelling during pregnancy and in the postpartum period^12,13^, modulation of stress responses, calcium transport, and reduction of anxiety^14–16^. It has been found that Prl provides neuroprotection against excitotoxicity in the hippocampi of lactating rats^17–21^. Recently, it has been demonstrated that prolactin treatment induces changes in the expression of the prolactin receptor and reduces the excitotoxicity generated by glutamate in primary hippocampal neuronal cultures^20^. Additionally, in the same model, neuroprotection has been associated with a decrease in the Ca^2+^ overload observed during kainic acid-mediated excitotoxicity, therefore promoting survival of hippocampal neurons^21^.

Considering the pleiotropic functions of Prl, recent efforts have been dedicated to elucidating the mechanisms of action of this hormone in different body tissues, such as duodenal epithelial cells^22^, mammary epithelial cells^23^ and pancreatic islets, by using transcriptomic approaches^24^. However, to the best of our knowledge, there is no information about Prl transcriptomic effects in the hippocampus.

Given that Prl regulates several brain processes, especially those that depend on the hippocampus, the present study aimed to elucidate the transcriptional effects of Prl in the hippocampus by means of transcriptomic analysis.

## Results

### Transcript abundance and differential regulation of gene expression induced by Prl treatment in the hippocampus

**Figure 1:**
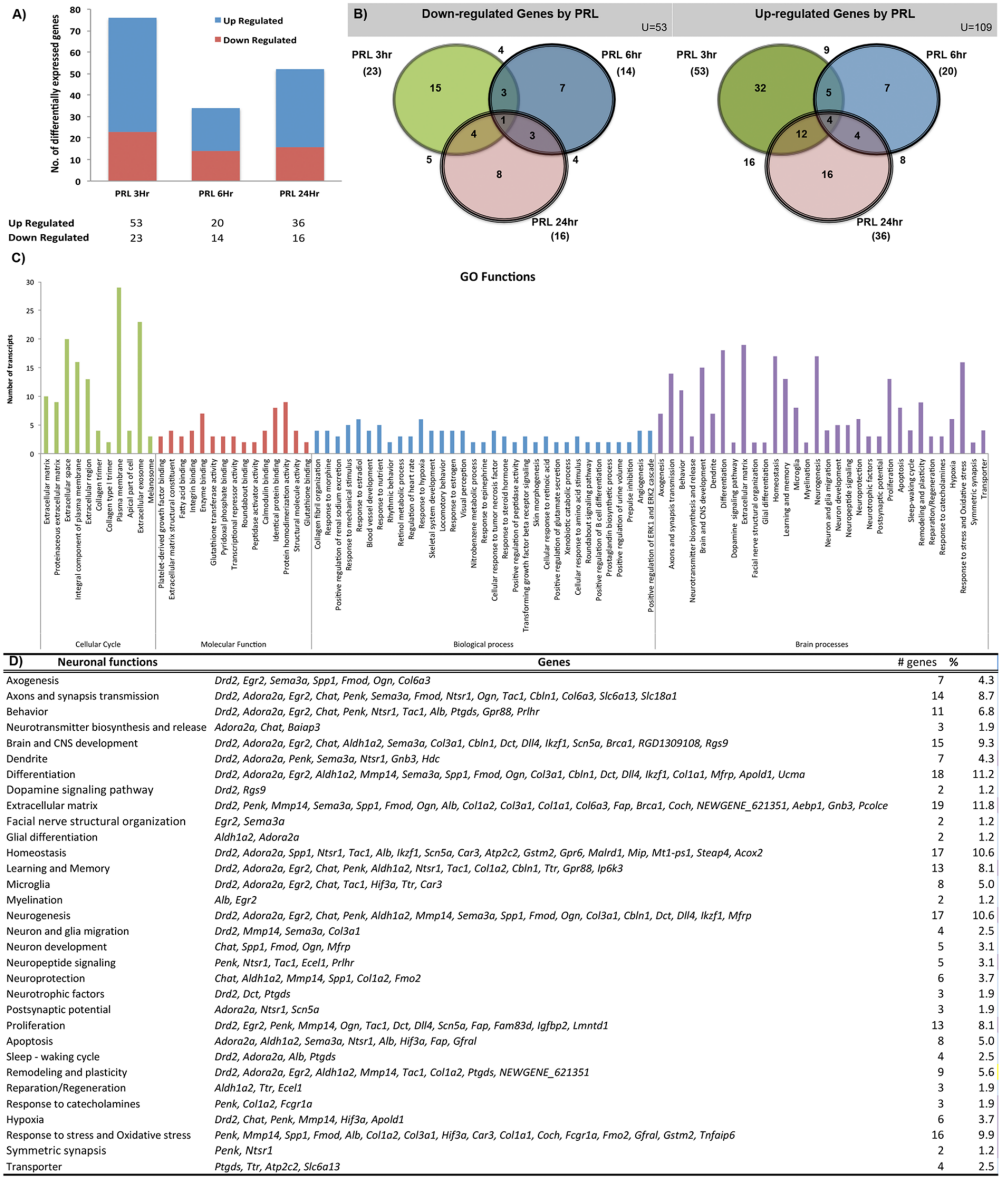
Prl induces changes in the expression of several genes involved in brain processes. (**A**) Summary of all the differentially expressed genes. (**B**) Venn diagram of the differentially expressed transcripts during time courses of Prl treatment. (**C**) Diverse categories of cell functions affected by Prl-regulated genes. All genes related to brain processes are shown in purple. (**D**) Numbers and percentages of Prl-regulated genes involved in brain processes.

A total of 365,050,464 reads, with a mean of 30.4 million reads (2 × 76 bp paired-end reads), were obtained for total RNA from each hippocampus sample. Up to 93.5% of the reads were successfully mapped to transcripts contained in the NCBI Rnor 6.0 genome reference (Supplementary Table S1). Figure 1A shows the changes in gene expression at 3, 6 and 24 h post Prl treatment. In particular, Prl treatment modified the expression of 162 genes in the hippocampus of ovariectomized rats throughout the 24 h period, where 109 genes were upregulated and 53 were downregulated in Prl-treated rats compared to control rats (Supplementary Table S2). The differentially expressed genes after 3 h of Prl treatment included 53 upregulated genes and 23 downregulated genes (Fig. 1A).

### Functional clusters of differentially expressed genes

During the time course of Prl treatment, persistent changes in gene expression were observed for five genes (Fig. 1B); three of the genes (*Fmo2, Egr2 and Apol1*) are involved in brain processes, and the other two were *Srpk3*, a serine/threonine protein kinase, and *Rhbdl2*, a trans-membrane serine protease (Fig. 1B). The functional classification of each gene modified by Prl treatment was obtained from the Gene Ontology (GO) and Ensembl databases. The genes induced by Prl at 3, 6 and 24 h were mainly associated with cell cycle regulation (28.0%, 36.1%, and 27.0%, respectively) and biological processes (23%, 44% and 44%, respectively); these genes represented more than 50% of the transcribed genes in each time period. Other biological processes were also represented, such as protein interactions, pathways and molecular functions (Fig. 1C and Supplementary Fig. S3A). In addition, few transcripts were related to disease processes, as shown in Supplementary Fig. S3A. In contrast, we detected several differentially expressed genes related to 32 brain processes (Fig. 1C), corresponding to 37.65% of all the differentially expressed genes. Interestingly, most of the genes differentially expressed after Prl treatment are involved in hippocampal functions such as learning, memory, microglial function, myelination, neurogenesis, neuroprotection, proliferation, remodelling, plasticity and hypoxia (Fig. 1C, purple bars, and Supplementary Fig. S3B). In particular, some of the induced genes (*Ddr2, Adora2a, Egr2, Penk, Chat*, and *Sema3a*) are involved in more than ten brain processes that are relevant to hippocampal functions (Fig. 1D).

Gene expression profiles related to brain processes changed throughout the time course of Prl treatment. Interestingly, the majority of genes were induced after 3 h of Prl treatment, which represents a primary response to Prl. In particular, 9 of 17 differentially expressed genes were involved in processes related to the extracellular matrix, differentiation, responses to oxidative stress, neurogenesis, proliferation, and brain and CNS development. However, after 6 h of treatment, we observed a reduction in the number of differentially expressed genes. The genes induced at this time point were related to brain and CNS development, axonal and synaptic transmission, learning and memory, and the extracellular matrix, and no more than four genes were involved in each process. Finally, after 24 h of Prl treatment, the most represented processes were learning and memory, homeostasis, brain and CNS development, and behaviour, and more than 8 genes were included for each process (Supplementary Table S4).

**Figure 2:**
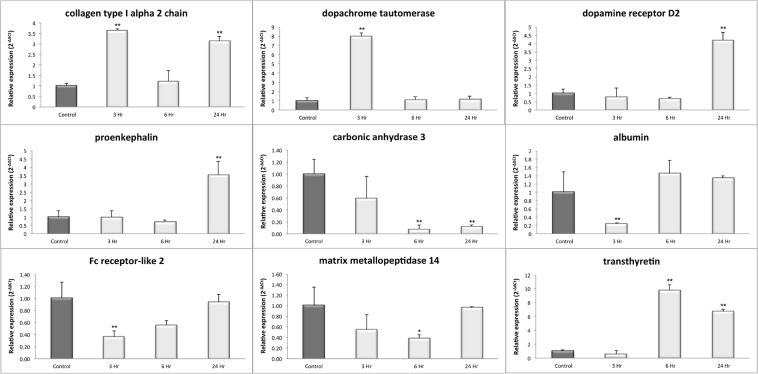
Validation of gene expression induced by Prl. RT-qPCR was used to validate nine genes with a Fc >2 by RNASeq analysis. Total RNA obtained from groups of animals treated with Prl (500 µg/kg) in a time course experiment for 3, 6 or 24 h or a control group (NaCl_2_, 0.9%) was used for RT-qPCR. RNA of the internal control gene *Hprt* was used to calculate the relative expression of each gene according to the 2^−ΔΔCt^ mathematic method. The light grey bars represent PRL treatment at 3, 6 and 24 h. The dark grey bars represent the expression control. The values are presented the mean ± SD, n = 3. *p < 0.05 vs. control; **p < 0.01 vs. control (paired t-test).

Since many differentially expressed genes were associated with several brain functions, the obtained results by RNASeq were validated for genes related to specific brain processes using RT-qPCR (Fig. 2 and Supplementary Tables S5, S6). The RT-qPCR analysis revealed the accuracy of the results obtained by RNASeq, and the expression results assessed by RT-qPCR are summarized in Fig. 2 and Supplementary Table S5.

**Figure 3:**
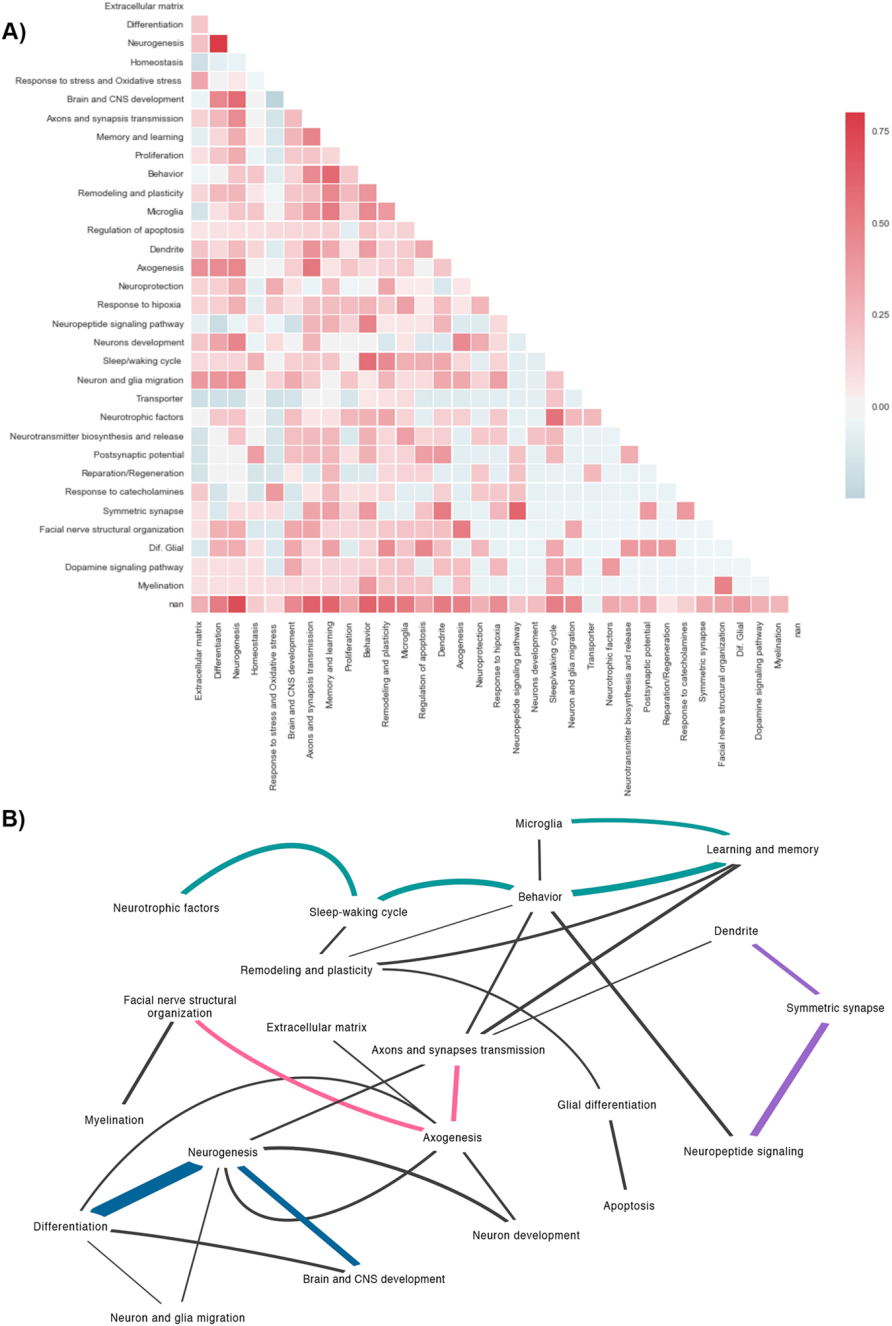
Correlation of Prl-induced gene expression associated with brain processes. Pearson correlation analysis between Prl-induced gene expression and induced functions was performed using Python 3.5 packages (matplotlib, numpy, matplotlib.pyplot, pandas networkx, and seaborn) and Cytoscape 3.6.1. (**A**) Heatmap of correlated functions. The strongest positive correlations and the strongest negative correlations between gene expression and brain processes are shown in red and blue, respectively. (**B**) A correlation network of main brain processes and genes is depicted. Main brain processes related to clusters of genes are shown in pink, cyan, blue and purple; the line thickness is proportional to the number of genes that are interacting.

Using GO data only for genes related to brain processes, we performed a correlation analysis between cell functions and gene expression (Fig. 3A), and a gene functional network was constructed (Fig. 3B). As observed in Fig. 3B, the most enriched network processes were those related to neurogenesis, differentiation, and CNS and brain development (blue lines); neurotrophic factors, the sleep–wake cycle, learning and memory, behaviour and microglia (cyan lines); facial nerve structural organization, axogenesis, and axonal and synaptic transmission (pink lines); and symmetric synapse, neuropeptide signalling and dendrite-related pathways (purple lines). Interestingly, this functional network in the hippocampus revealed that Prl induced genes related to sleep–wake cycle regulation, glial functions, myelination, and neuronal differentiation, which have previously been reported in other brain areas^3^. On the other hand, other processes, such as glial differentiation, axogenesis, synaptic transmission, postsynaptic potential, facial nerve structural organization, and neuronal and glial migration, amongst others, are new processes associated with Prl effects in the hippocampus.

A representative element network analysis that includes a summary of relations between genes and brain processes regulated by Prl is depicted in Supplementary Fig. S7. Importantly, as observed in this Figure, six genes (*Drd2, Sema3a, Egr2, Chat, Penk and Adora2a*) were involved in ten or more processes regulated by Prl. In this analysis, ten brain processes were highly regulated by Prl, represented by more than ten genes. These genes are related to behaviour, learning, memory and neurogenesis, which are classic hippocampal functions. However, other processes observed in this study, such as brain and CNS development, differentiation, remodelling and plasticity, have not been previously associated with Prl actions in the hippocampus.

### Immunodetection analysis indicates that Prl modified the expression of microglia and neuronal proteins involved in hippocampal plasticity

**Figure 4:**
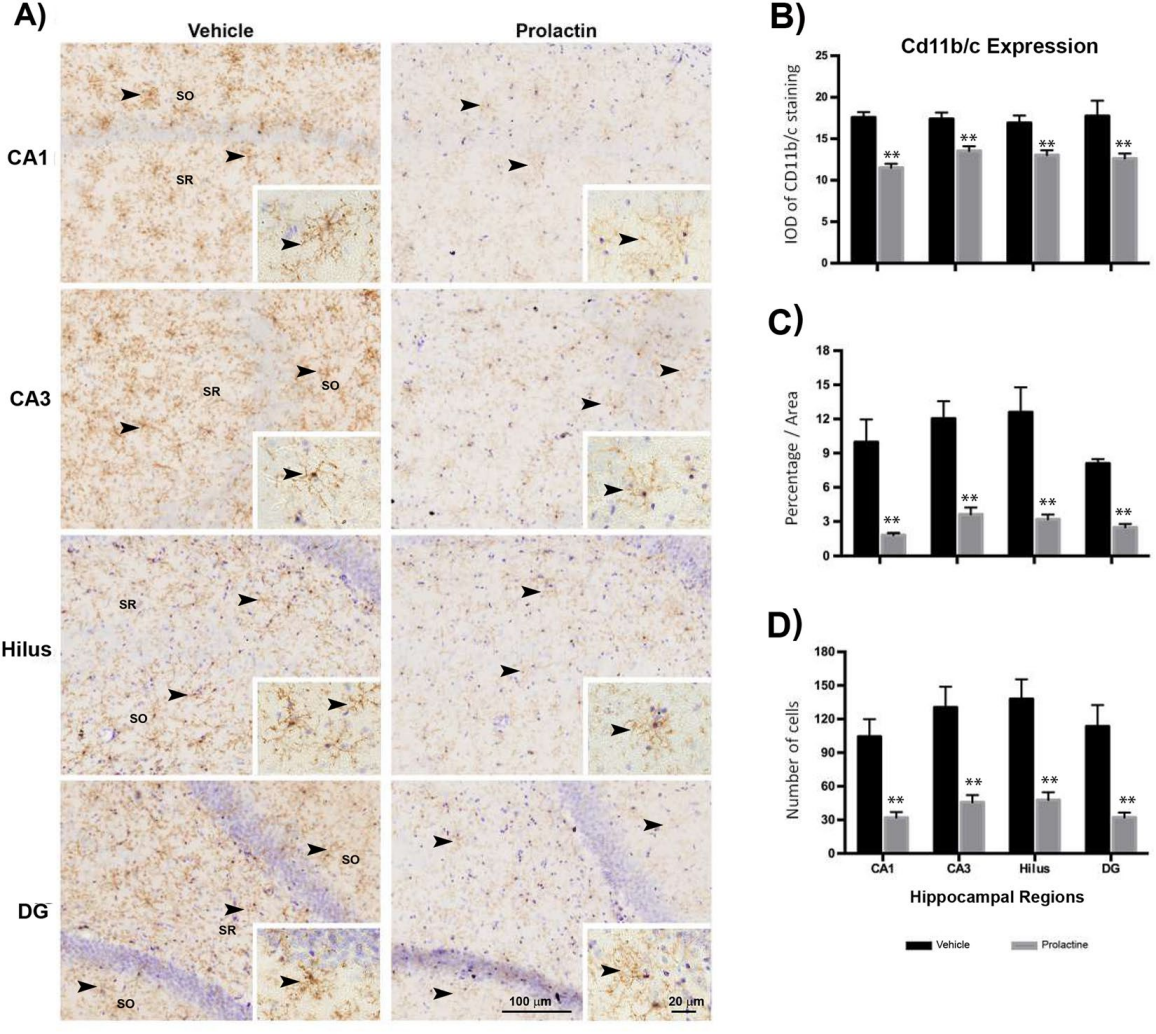
Prolactin induces attenuation of microglial activation in the hippocampus. Immunohistochemistry for Cd11b/c was performed in the hippocampus. (**A**) Immunostaining of Cd11b/c in hippocampal subfields (CA1, CA3, hilus and DG) from animals treated with vehicle and Prl is shown with details of microglial morphology (inset). Quantitative analyses of (**B**) Cd11b/c expression, (**C**) the areas covered by microglia, and (**D**) the numbers of particles expressing Cd11b/c in each hippocampal region are also shown. Significant differences were lower than p ≤ 0.001 by t-test (**). Scale bars = 100 µM and 20 µM.

To explore the effect of Prl treatment on hippocampal processes such as those related to microglia, axons and synaptic transmission that were observed in our GO and network analysis, we selected proteins related to these functions. First, to determine the effect of Prl treatment on hippocampal microglia, we performed immunodetection of Cd11b/c, a b-integrin marker of microglial cells, in hippocampal tissue (the relations of this gene with other genes detected in our transcriptomic analysis are depicted in Supplementary Table S8). As shown in Fig. 4A, prolactin treatment had a notable effect on microglial cells of all hippocampal areas (CA1 and CA3). Cd11b/c expression was significantly decreased in the prolactin group compared to the control group, and the microglial projections were thinner (see inset in Fig. 4A). The analysis of cell immunolabelling density (area coverage, intensity of protein expression and number of particles expressing the protein) showed significant decreases in Prl-treated animals compared to control animals (Fig. 4B–D, bar graphs).

**Figure 5:**
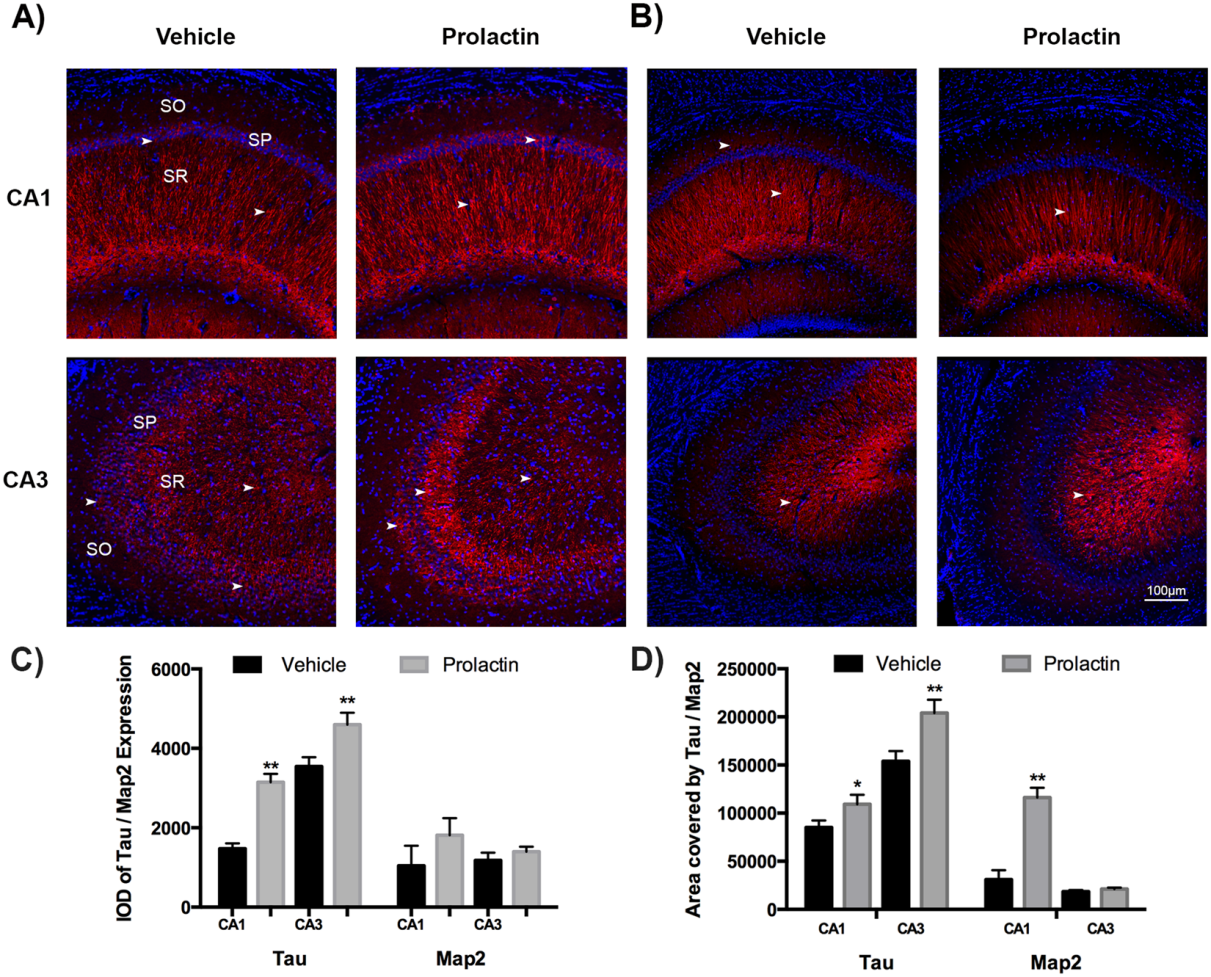
Prolactin treatment modifies the cellular location and content of Tau and Map2 proteins in the hippocampus. Immunohistochemistry against total Tau and Map2 proteins in hippocampal tissues was performed. (**A**) Tau protein detection in hippocampal subfields (CA1 and CA3) in animals treated with vehicle compared with those treated with Prl. Changes in SO and SR were detected. (**B**) Map2 protein detection in the hippocampus. Major changes were detected in the somas of pyramidal cells (stratum oriens: SO, stratum pyramidale: SP, and stratum radiatum: SR). Nuclei were counterstained with DAPI (blue). Scale bar = 100 µM.

Moreover, the expression of neuronal proteins, such as Tau, Map2 and Synaptophysin (Syp), was assessed (these proteins were also related to many genes induced in our transcriptomic analysis, as shown in Supplementary Table S8). Tau and Map2 are mainly involved in microtubule stability and cargo transport, while Syp is related to synaptic functions. Tau expression presented a significant increase in CA1 in neuronal somas, and its expression had greater intensity and area in neuronal projections of Prl-treated animals than in those of vehicle-treated animals (Fig. 5A,C,D). However, in CA3, this protein was localized in neuronal somas but was mainly localized in neuronal projections to the SR and was redistributed after PRL treatment, localizing with more intensity near the projections of neuronal somas (Fig. 5A). Interestingly, Map2 expression was localized in the projections of the stratum pyramidale, and no differences in intensity were detected between treatments; however, in CA1, the area covered was increased in the Prl group compared to the control group (Fig. 5B–D).

**Figure 6:**
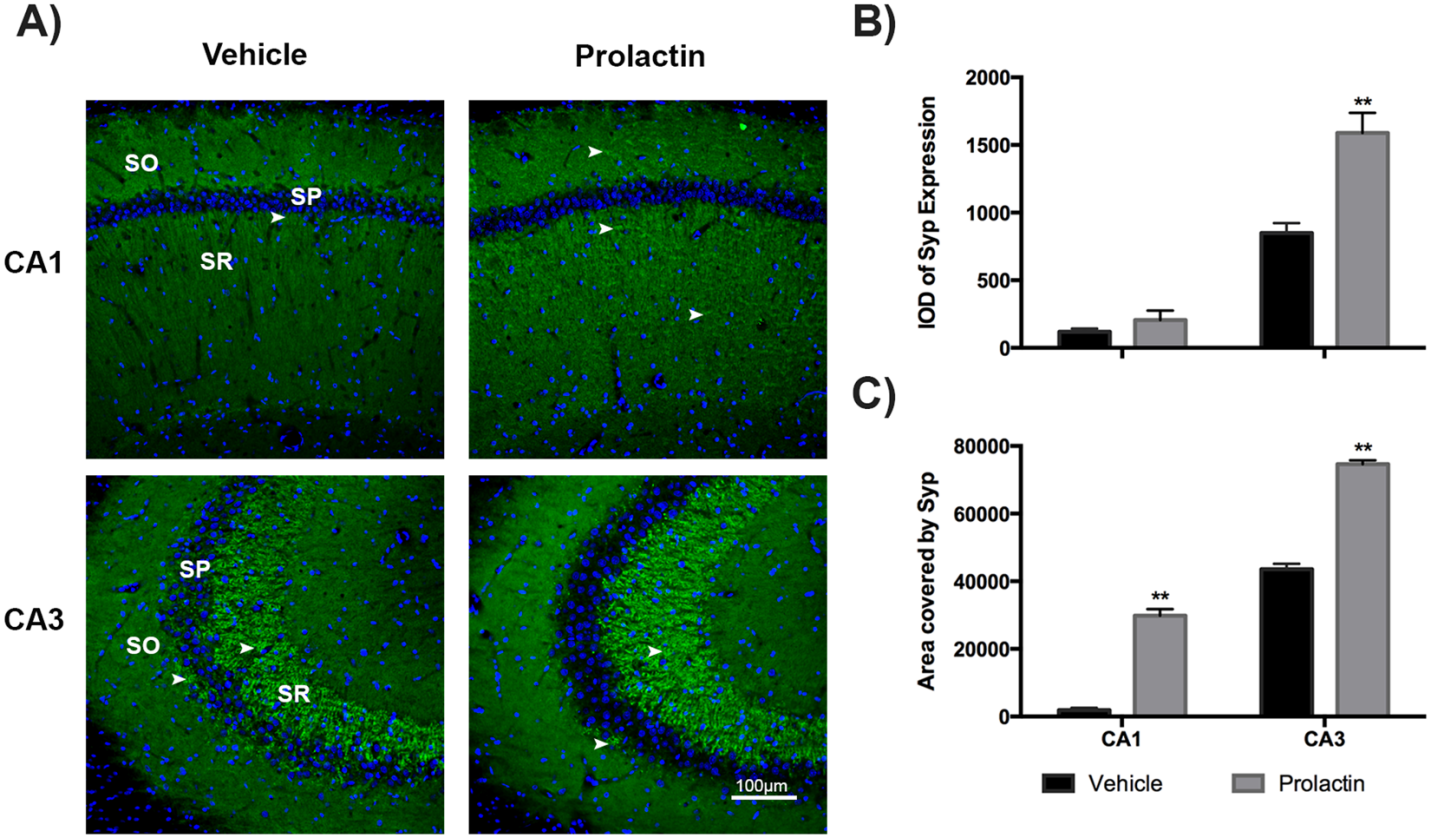
Prolactin treatment modifies the cellular location and expression of Syp protein in the hippocampus. Immunohistochemistry against total Syp protein in hippocampal tissues was performed. (**A**) Syp protein detection in hippocampus subfields (CA1 and CA3) in animals treated with vehicle compared with those treated with Prl (stratum oriens: SO, stratum pyramidale: SP, and stratum radiatum: SR). Nuclei were counterstained with DAPI (blue). Scale bar = 100 µM.

Figure 6A–C depicts Syp expression and quantitation. As observed, Syp expression in CA1 was almost absent in control animals, while Syp expression in the same region was slightly but not significantly increased in Prl-treated animals. In addition, in the CA3 region, a significant and marked increase in Syp expression was observed in PRL-treated animals compared with control animals that was localized in neuronal projections, mainly in the SR.

## Discussion

The expression of the *Prl* gene and the Prl receptor (PrlR) have been described in different brain areas. Although PrlR protein has been found in the brain, the details of Prl protein expression are still controversial^3^. Furthermore, the effects of Prl on mental processes and behaviour in mammals, including humans, have been largely recognized. However, to the best of our knowledge, there is no information about Prl effects at the transcriptional level in the brain, particularly in the hippocampus.

The results of the current study indicate that Prl administration induces transcriptional activity in the hippocampi of OVX female rats. Prl effects at the transcriptional level were analysed in a time course experiment throughout 24 h after Prl administration. Among the time periods, the greatest number of differentially expressed genes was observed after 3 h of Prl treatment, and the differentially expressed genes were related to restructuring, remodelling, proliferation and neuronal development processes, indicating neuronal plasticity. Furthermore, after six hours of treatment, the processes in which Prl was involved were related to cognitive functions, synapses and neuronal development. In addition, 24 h after Prl administration, the activated processes were related mainly to mental functions such as learning, memory and behaviour, indicating a possible sequential role of Prl regulation in hippocampal processes and functions; furthermore, it is notable that PRL principally exerted a primary transcriptional effect followed by a secondary transcriptional response. Indeed, of all the genes that were differentially expressed throughout the 24 h period after Prl treatment, 37.65% were related to brain processes (Fig. 1 and Supplementary Fig. S3). Importantly, to explore the brain processes activated by Prl, we studied the expression of proteins related to microglia and neuronal plasticity. Interestingly, treatment with Prl modified the expression and location of the microglial activation protein Cd11b/c as well as neuronal proteins such as Tau, Map2 and Syp, all of which are involved in neuronal plasticity.

Although Prl was discovered at the beginning of the last century, there is little information on the molecular mechanisms through which Prl regulates neuronal functions. It is known that Prl regulates specific neuronal circuits and participates in many brain functions, including maternal behaviour, energy balance and food intake, sleep, anxiety, neurogenesis, and migraine and pain, among others^11,14,25–30^. A recent review by Patil and coworkers^16^ highlighted the effects of Prl in neuron excitability, describing the critical effects of this hormone on membrane receptor potential thresholds and/or neurotransmission efficiency. The authors concluded that the action of Prl in neurons is mainly exerted through regulation of neuronal excitability and channels, such as TRPV1, TRPA1, and TRPM8^16^. However, the precise gene expression changes associated with these important functions have not been explored. Our results provide the molecular basis for defining the functional impact of Prl in the hippocampus.

In this study, transcriptomic analyses indicated that Prl induces the expression of different sets of genes involved in brain processes related to learning, behaviour, memory, neuroprotection, neurodevelopment, neurogenesis, remodelling, plasticity, and sleep/waking regulation, among others (Figs 1, 3). These processes have been previously found to be related to Prl functions in this brain area^3^. Furthermore, the data analyses indicated that other important processes, such as glial differentiation, axogenesis, synaptic transmission, postsynaptic potential, facial nerve structural organization, and neuronal and glial migration, might also be regulated by Prl in the hippocampus (Fig. 3, Supplementary Fig. S7).

We were able to detect six genes induced by Prl (*Drd2, Sema3A, Egr2, Chat, Penk* and *Adora2a*), and importantly, each of these genes was associated with the regulation of more than ten brain functions and processes (Fig. 3, Supplementary Fig. S7) related to axons as well as synaptic transmission and microglial regulation. Considering the characteristics of these genes, we think that these genes may play major roles in hippocampal functions and that they deserve to be further studied.

Other genes, such *Ador2a, Car3, Chat, Drd2, Egr2, Hif3a, Notch, Penk, Tac1* and *Ttr*, which were also differentially expressed after Prl treatment, are involved in maintaining microglial functions. Interestingly, these genes have been reported to be altered in neuroinflammation, neurodegenerative diseases, and psychiatric disorders^31–39^. It is possible that genes related to microglial functions induced by Prl might play roles in neuro-immunomodulation in the hippocampus or in preventing neuronal cell damage. This idea is consistent with previous reports that Prl confers neuroprotection in excitotoxicity models^3,17,18,40^. It is important to note that microglia have been related to either neurodegenerative or neuroprotective processes depending on their activation state. Whether prolactin neuroprotection is a consequence of direct effects of Prl on microglial cells or to Prl-mediated inhibition of a non-classic excitotoxicity pathway has yet to be elucidated.

In this regard, our results demonstrated that Prl modified microglial activation after 24 h of Prl treatment, which was shown by a significant decrease in the expression of the Cd11b/c marker (Fig. 4). This observation supports and extends the idea that Prl may modulate microglial functions, which was previously reported by Möderscheim *et al*. in a study indicating that Prl is primarily involved in a gliogenic response during recovery from cerebral injury^41^. Many other functions of glial cells have been reported; for example, microglia engulf and remodel developing and mature synapses^32^. Moreover, microglia contribute to good connectivity during normal assembly of brain circuits^34^. Considering that the Prl receptor is present in primary cell cultures of hippocampal neurons^20^, it is important to determine whether Prl may participate in these processes during hippocampal development and in the adult brain.

Furthermore, it has been widely reported that microglia play important roles in several physiological processes not related to pathological conditions, such as axogenesis, neurogenesis and synaptogenesis; these processes are also affected by Prl^42^. Further studies are required to establish whether microglial activation, as well as morphological changes in microglia, are related to the neuroprotective effect induced by prolactin. In another study, long after rats were ovariectomized, Prl binding sites and serum prolactin levels were found to be reduced in many brain areas^43^; in addition, long-term ovariectomy causes cognitive dysfunction associated with neurogenesis, synaptic plasticity and immune modulation and also induces changes in the hippocampal transcriptome^44^. In our study, we showed that prolactin treatment in short-term ovariectomized rats reduced microglial activation and caused morphological changes in microglial cells (Fig. 4), which may be related to microglial functional recovery. However, more investigation will be required to explore this effect.

On the other hand, it has been reported that upregulation of the adenosine A2A receptor protein (Adora2a) in neurons is associated with synaptic impairment^45^. Interestingly, increased Adora2a gene expression has been associated with neurogenesis and hippocampal volume in Alzheimer patients^46^, which is a very interesting finding that should be further explored.

The remarkable modification of gene expression induced by Prl related to hippocampal processes led us to evaluate proteins involved in some of these brain processes. We performed a series of experiments to detect changes in neuronal proteins, such as Tau and Map2, that are involved in axonal transport and synaptic plasticity. After Prl treatment, these proteins showed marked changes in their expression and localization (Fig. 5). These neuronal proteins have been largely recognized as modulators of neuronal plasticity, thus suggesting that Prl has an effect in neuronal plasticity. We also evaluated a neuronal protein involved in synaptic transmission, Syp. Indeed, Prl treatment also markedly modified the expression and neuronal localization of this protein, as shown in Fig. 6. Thus, the changes observed in this protein, namely, increased expression and changes in cellular localization after Prl treatment, suggest that this protein plays a role in the modulation of synaptic function and reinforces the idea that this may be a Prl function in the hippocampus. This proposal is consistent with our recent report, which revealed that Prl induced an increase in the expression of vesicular glutamate transporter 1 in the hippocampus concomitantly with an increase in the expression of PrlR^47^. However, the possible effects of Prl on excitatory and inhibitory synaptic transmission require further investigation.

On the other hand, hippocampus-dependent plasticity has been shown to be related to genes such as *Drd2*, which plays a major role in the regulation of hippocampal learning and memory^48^ as a modulator of microglial function during neuroinflammation^49^ and in preventing damage by reducing inflammation^50^. Interestingly, *Drd2* gene expression was highly regulated by Prl in this study. Furthermore, other differentially expressed genes regulated by Prl, such as Ntsr1, have been shown to contribute to different neuronal functions. Ntsr1 plays a role in the induction of long-term potentiation at granular cell synapses^51^. Interestingly, Cbln1 can induce changes in dynamic axonal structure during cerebellar synapse formation^52^. Furthermore, Sema3a regulates neuronal morphogenesis through polarization of dendrite/axon formation in hippocampal neurons in primary cell cultures^53^. Whether these genes induced by Prl in the hippocampus play similar roles in other brain areas is a question that needs to be further explored.

Surprisingly, we found that Prl modulates many genes of the extracellular matrix, which have been reported to prevent ageing^54^. In particular, genes such as *Col1a1 Col1a2, Fmod, Ptgds, and Aldh1a2* are induced by exercise training and have been found to prevent hippocampal ageing in an Alzheimer’s disease model of animals with premature ageing. Remarkably, we found that all of the above genes were induced in the hippocampus by Prl (Fig. 1D and Supplementary Table S2), suggesting a possible important role for Prl in preventing ageing in the hippocampus. However, further studies are required to assess this possibility.

In addition, some genes found in the current study display many brain functions, such as the Penk gene, which is induced during neuronal excitation in the hippocampus and is involved in neuroprotection against kainic acid in a ketogenic protective diet^55^. This gene is also overexpressed in dopaminergic tuberoinfundibular neurons of pregnant, pseudopregnant, lactating and aged rats, suggesting that this gene is regulated by Prl, as confirmed by the present study^56^. Furthermore, stress increases Prl synthesis, which subsequently induces *Penk* expression in the parvocellular subset of the paraventricular nucleus, suggesting that Prl regulation of Penk expression participates in reducing damage induced by stress^57^. However, the complete function of this gene in the hippocampus requires further elucidation.

In the field of psychiatry, the adverse effects of Prl remain under debate. Treatment with psychiatric drugs to manage schizophrenia and depression is frequently associated with hyperprolactinemia (HPL), suggesting the negative effects of Prl in these patients^58,59^. In contrast, in a recent study, it was shown that many patients with a first episode of psychosis presented HPL before treatment and presented elevated rates of HPL over the course of the illness^60^. Further studies are required to determine whether HPL alters brain processes during the course of disease and treatment or whether HPL may be involved in a compensatory mechanism to maintain brain tissue homeostasis in these patients. The finding of this study that Prl seems to fine-tune gene expression regulation in the hippocampus opens up a novel field of research on psychiatric diseases.

In conclusion, the overall results of this study support and extend the idea that Prl is a neuroactive hormone that induces transcriptional effects in several sets of genes that may be involved in several hippocampal functions, including neuronal plasticity.

## Materials and Methods

### Animals

Adult virgin female Wistar rats (250–300 g) were individually housed under a controlled temperature and photoperiod (12:12 h light:dark cycle, lights on at 06:00), with food and water available *ad libitum*. To produce ovariectomized (OVX) rats, the ovaries were surgically removed under anaesthesia (0.2 ml/100 g of body weight, intraperitoneal administration of a cocktail of 87.5 mg/kg ketamine and 12.5 mg/kg xylazine, Cheminova de México, México), and Prl treatment was started 2 weeks after surgery. Three rats from each group were randomly assigned to each of four groups: the vehicle group, the Prl 3 group, the Prl 6 group, and the Prl 24 h group. The Institutional Animal Care and Use Committees of the School of Chemistry at the National Autonomous University of Mexico approved all experimental protocols. The animals were handled in accordance with the National Institutes of Health Guide for the Care and Use of Laboratory Animals and the Official Mexican Guide of the Ministry of Agriculture (SAGARPA NOM-062-Z00-1999) published in 2001. All efforts were made to minimize suffering and the number of animals used.

### Treatments

OVX rats were treated once, intraperitoneally, with a single dose of Prl from the sheep pituitary (Sigma-Aldrich, L6520) (500 µg/kg in 100 µl of 0.9% sterile saline solution) or with a control solution (100 µl of 0.9% sterile saline solution). The groups were as follows: one control group treated with vehicle and three Prl groups treated for 3, 6 and 24 h.

### RNA extraction

For RNA extraction, the hippocampi were dissected from the animals. Total RNA was extracted with an RNeasy Plus Mini Kit Qiagen (Qiagen, 74004) following the manufacturer’s instructions. The RNA was quantified, and the integrity was assessed with both a NanoDrop (model ND-1000) and an Agilent 2100 Bioanalyzer prior to library preparation.

### RNASeq

Total RNA (2 µg) was used for library preparation, according to the manual of the TruSeq Stranded mRNA Library Prep Kit (Illumina, 20020595). Libraries with fragments of 250–450 bp were sequenced as 76 base pair paired-end reads by the Unidad Universitaria de Secuenciación Masiva y Bioinformática (UNAM) using a NextSeq. 500 platform (Illumina). A total of 365,050,464 reads and a mean of 30.4 million reads (2 × 76 bp paired-end reads) were obtained for the total RNA from each hippocampus sample.

### Data analysis

The sequence output in FastQ format was screened for quality using FastQC software provided by Babraham Bioinformatics (http://www.bioinformatics.babraham.ac.uk/projects/fastqc/), and alignment was carried out with the rat genome downloaded from NCBI (GCF_000001895.5_Rnor_6.0_genomic.fna.gz) using the Smalt program with its default values. For all the samples, coverage calculation by gene was carried out with the BamTools BED coverage tool using the NCBI GFF file GCF_000001895.5_Rnor_6.0_genomic.gff.gz, and an alignment exceeding 93% was obtained after filtering for only genes. The matrix result was introduced into IDEAmex (http://zazil.ibt.unam.mx/ideamex), and NOISeq, DESeq, EdgeR and DESeq. 2 were for differential expression analysis using an FDR (false discovery rate) of 0.05 and a log2(fold change) of 1. Genes with differential expression ≥2-fold or ≤2-fold were included in the analysis.

Correlations and networks were analysed with Pearson correlation analysis between Prl-induced gene expression and gene functions using Python 3.5 packages (matplotlib, numpy, matplotlib.pyplot, pandas networkx, and seaborn) and Cytoscape 3.6.1.

### Functional clustering

To perform functional annotation clustering, the gene IDs for the differentially expressed genes (both upregulated and downregulated) were uploaded into the DAVID v6.7^61^; the gene IDs used were the official names converted to symbols, which are compatible with the DAVID.

### RT-qPCR

Validation was performed to verify the expression of selected genes, the RNA used for RNASeq. The genes subjected to validation were Ttr, Drd2, Car3, Gfral, Col1a2, Dct, Mmp14, Egr2 and Fcrl2. The RNA was quantified on a NanoDrop ND-1000 (Thermo Scientific, USA), and cDNA was reverse-transcribed using a Reverse Aid First Strand cDNA Synthesis Kit (Thermo Scientific, USA) according to the manufacturer’s instructions with polyT primers. RT-qPCR was performed using TaqMan® Fast Universal PCR Master Mix (Applied Biosystems) under the following conditions: 95 °C for 20 s followed by 40 cycles of 95 °C for 1 s and 60 °C for 20 s. RT-qPCR was performed using a StepOne Plus Real Time PCR system (Applied Biosystems, USA). All run results were normalized to the *Hprt* control for analysis. Sequence Detection Software 1.3 (Applied Biosystems) was used for data analysis. The comparative CT method (2^−ΔΔCT^) was used to calculate the relative changes in target gene expression. The average and standard deviation of 2^−ΔΔCT^ were calculated for the three independent experiments. Applied Biosystems supplied the probes for all genes. All assays were purchased from Applied Biosystems (see additional file Supplementary Table S6 for the primer sequences and amplicon lengths).

### Transcardial perfusion fixation of the CNS

For tissue extraction, rats were anaesthetized with pentobarbital (Sigma-Aldrich, St. Louis, MO) and perfused transcardially with 250 ml of PBS (0.1 M; pH 7.4) followed by 250 ml of 4% paraformaldehyde in PBS (pH 9.5, 10 °C). The brains were removed, post-fixed in the same fixative overnight and cryoprotected with 20% sucrose for 2–3 days at 4 °C. Coronal sections (30 μM) were cut through the dorsal hippocampus on a freezing microtome, and the serial cuts were collected and stored in cryoprotectant solution (30% ethylene glycol and 30% glycerol in PBS) at −20 °C. Three tissue sections were employed for each round of staining. Before performing all procedures, free-floating tissues were rinsed three times over a period of 10 min in PBS buffer.

### Immunohistochemistry

Immunoreactivity for Cd11b/c (Abcam, ab1211) was detected using a conventional avidin–biotin–immunoperoxidase technique^62^. The tissues were labelled with a cell type-specific monoclonal mouse antibody against Cd11b/c (1:500). Floating tissues were treated with 3% hydrogen peroxide for 10 min to quench endogenous peroxidase activity, rinsed three times in PBS and then incubated in 1.0% sodium borohydride for 6 to 8 min to reduce free aldehydes. The tissues were then incubated with blocking solution (5% BSA, 2% goat or rabbit serum, 1% Triton X-100 in PBS) for 1 h to decrease non-specific labelling. The tissues were later incubated with primary antibodies at room temperature overnight. After washing, the primary antibodies were detected with a biotinylated secondary antibody (1:1000; Santa Cruz Biotechnology, CA) and an avidin/biotin system (VECTASTAIN Elite ABC kit, Vector Laboratories, Burlingame, CA). Images were collected with an Olympus microscope and analysed using ImageJ free software.

### Immunofluorescence

Labelling for Syp, Map2 and Tau proteins was performed on free-floating brain tissues. The tissues were treated with 0.1% sodium citrate at 4 °C for 30 min, incubated in 0.1% Triton X-100 in PBS for 30 min at room temperature and blocked with blocking solution (5% bovine serum albumin, 2% normal goat serum, 0.1% Triton X-100 and 0.05% Tween 20 in PBS) for 1 h before incubation with the primary antibodies. The tissues were incubated overnight with monoclonal mouse anti-Syp (1:500; Santa Cruz Biotechnology SC-123737), mouse anti-Map2 (1:500; Santa Cruz Biotechnology SC-51669) and mouse anti-Tau (1:1000; Cell Signaling 4019) antibodies in 50% blocking solution with PBS. The tissues were rinsed three times in PBS for 10 min each and incubated in the dark with Alexa 647-conjugated anti-mouse IgG (1:500, Thermo Fisher Scientific) secondary antibodies for 1 h. After washing, the nuclei were stained for 5 min in a 1 µL/mL 6-diamidino-2-phenylindole-dihydrochloride (DAPI) solution (Boehringer Mannheim GmbH, Mannheim, Germany), and the tissues were coverslipped with fluorescence mounting medium (DAKO, Santa Clara, CA, USA). The control tissues were treated in the same way but without the primary antibodies. Images were collected with a Nikon confocal microscope.

### Statistical analysis

All numerical data are expressed as the mean ± SD and were calculated from three independent experiments. Statistical analysis of each data series was performed using one-way analysis of variance (ANOVA) followed by a post hoc analysis with a Newman-Keuls test and paired T-tests using Graph Prism 7 (GraphPad Software, Inc., La Jolla, CA, USA). A p-value ≤ 0.05 was considered to indicate a significant difference. To rank the abundance (enrichment) of genes and functional clusters, an enrichment score was calculated as the geometric mean of all the enrichment p-values for each annotation term associated with the gene in the brain functions^63^. Higher enrichment scores were indicative of a greater response of the gene members to the specific treatment, and in the case of functional clusters, a cut-off of 1.3 was used, as recommended by Huang *et al*.^61^. The p-value was determined using a modified Fisher’s exact test with a default cut-off of 0.1. Smaller p-values indicated greater significance of the enrichment of individual gene terms.

## Supplementary information

Table S1, Table S2, Table S4, Table S5, Table S6, Table S8

Figure S3, Figure S7

## Data Availability

Accession number: GSE119435.
