# Supplementary material for: Transcriptomic analysis reveals new hippocampal gene networks induced by prolactin
Source: Sci Rep. 2019 Sep 24;9:13765. doi: 10.1038/s41598-019-50228-7 (PMC6760160; doi:10.1038/s41598-019-50228-7)
Supplement: Supplementary file 2 — Figure S3, Figure S7 [file 41598_2019_50228_MOESM2_ESM.docx]

**Transcriptomic analysis reveals new hippocampal gene networks induced by prolactin**

**Abbreviated title:** Prolactin induce hippocampal gene networks

**Authors:** Erika Alejandra Cabrera-Reyes^1^, América Vanoye–Carlo^2^, Mauricio Rodríguez-Dorantes^3^, Edgar Ricardo Vázquez-Martínez^1^, Nadia Alejandra Rivero-Segura^5^, Omar Collazo-Navarrete^4^, and Marco Cerbón^1*^

**Supplementary legends**

**Table S1. RNASeq reads coverage.** Reads generated from RNASeq in the control group, and Prl-treated groups (3, 6 and 24 h) were aligned to the rat genome (Rnor 6.0).

**Table S2. Up and down regulated genes by Prl.** The matrix generated by the alignment of reads was introduced to the IDEAmex for differential expression analysis using NOISeq, DESeq and EdgeR with an FDR of < 0.05 and a Fc ≥ 2 or ≤ 2. Official gene symbol, gene name and FoldChange (Fc) are shown in columns 1, 2 and 3-5, respectively.

**Figure S3. Enrichment and Functional analysis of Prl regulated genes.** Differentially expressed genes after Prl treatment respect to vehicle group were analyzed using DAVID v6.7. (A) Within the GO categories, the gene products from biological processes and cell cycle were the most enriched by Prl treatment. B) Changes in the number of genes induced by Prl in a 24 h period related to brain processes are described; 3 h (blue), 6 h (red) and 24 h (purple).

**Table S4. Brain processes and functions.** Number of genes differentially expressed (#) after 3, 6 and 24 h of Prl treatment respect to vehicle group.

**Table S5.** **RNASeq and RT-qPCR data.** Fold change (Fc), p-value and brain processes of the selected genes after 3, 6 and 24 h of Prl treatment compared with to the vehicle group.

**Table S6.** Prl regulated genes validated by RT-qPCR and molecular characteristics of the nine validated genes.

**Figure S7. Network of brain processes and its association with Prl regulated genes expression.** The network analysis was performed by means of Python 3.5 packages (matplotlib, numpy, matplotlib.pyplot, pandas networkx, seaborn and Cytoscape 3.6.1 packages). Purple nodes represent brain processes influenced by Prl, whereas cyan nodes represent genes whose expression is modified by Prl; the number of genes and related functions are depicted by color intensity, with a stronger intensity representing a greater number of genes.

**Table S8.** Proteins selected for immunodetection and its relation with Prl induced genes detected in the global transcriptome.

**Figure S3.**


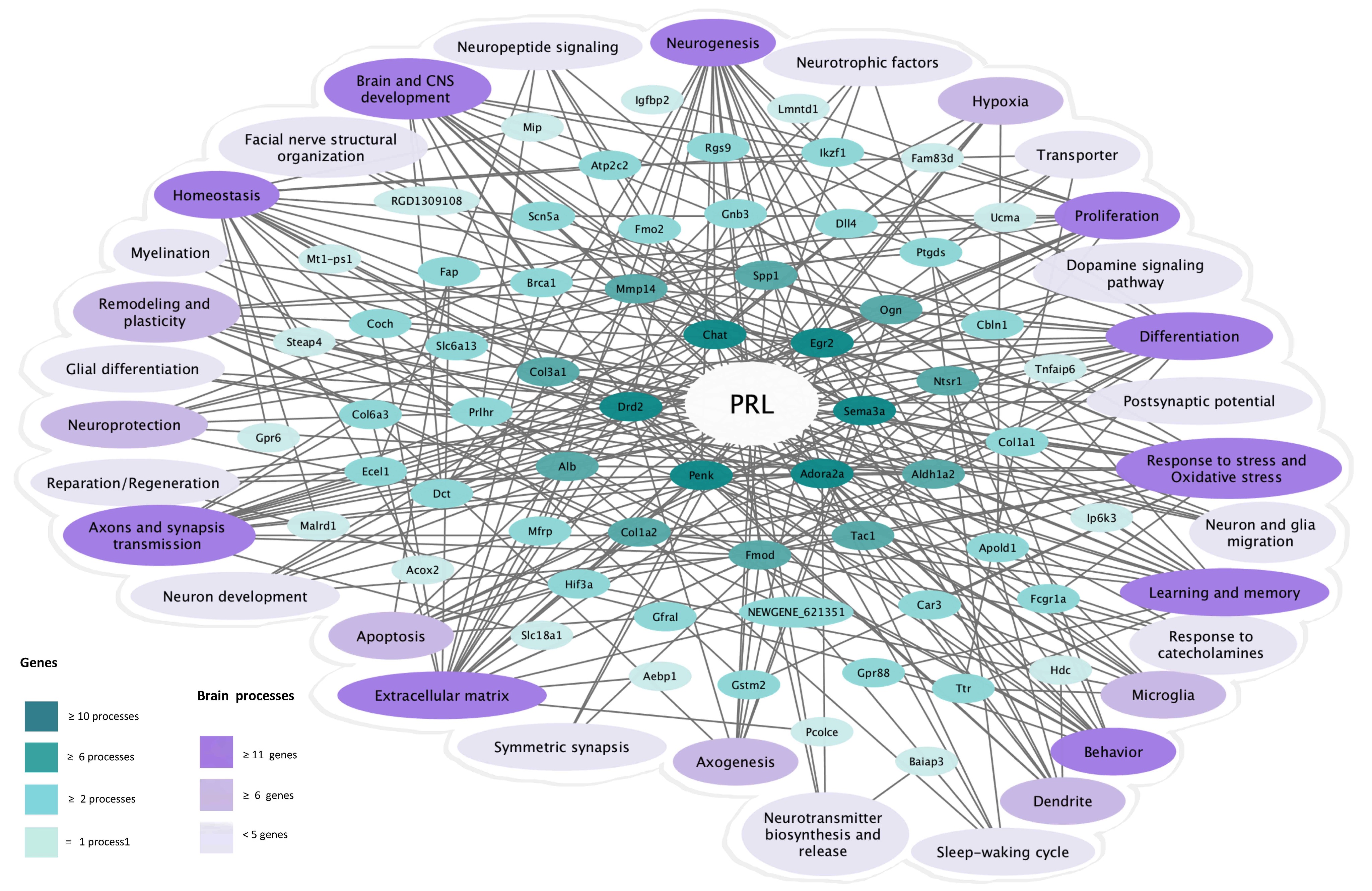


**Figure S7**
